# 

**Published:** 1890-10

**Authors:** 


### CONTENTS FOR OCTOBER, 1890.

ORIGINAL COMMUNICATIONS.

Insanity as a Symptom of Bright's Disease. By ALICE BENNETT, M. D. .... 129
Kraurosis Vulvæ. By CHARLES N. SMITH, M. D. .... 160

SOCIETY PROCEEDINGS.

Buffalo Medical and Surgical Association .... 164

PROGRESS IN MEDICAL SCIENCE.

Sanitation. By JAMES W. PUTNAM, M. D. .... 173

CORRESPONDENCE.

Letter from J. P. and J. H. .... 176

EDITORIAL.

The Hospital System of Sweden .... 180
Medical Department of Niagara University .... 182
The Medical Department of thé University of Buffalo .... 183
Census of Hallucinations .... 183
American Gynecological Society .... 184
The American Association of Obstetricians and Gynecologists .... 186
Dr. Eleanor McAllister .... 186

PERSONAL.

Dr. Wm. C. Krauss — Dr. Dagenais .... 187

Dr. Stockton — Dr. H. Y. Grant .... 186

BOOK REVIEWS.

Mineral Springs and Health Resorts of California .... 187
A Text-Book of Practical Therapeutics .... 188
The Pharmacology of the Newer Materia Medica .... 190
Railway Surgery .... 191
Essentials of Refraction and the Diseases of the Eye .... 191
Philosophy in Homeopathy .... 192

